# Whole Abdominal-Pelvic Radiotherapy in the Management of Primary Ewing Sarcoma of the Peritoneal Cavity

**DOI:** 10.7759/cureus.455

**Published:** 2016-01-11

**Authors:** Osmar Barbosa Neto, Aurelie Garant, Shakir Shakir, Josee Brossard, Perrine Garde-Granger, Carolyn Freeman

**Affiliations:** 1 Radiation Oncology, McGill University Health Centre; 2 Pediatric Hematology/Oncology, University of Sherbrooke; 3 Department of Pathology, University of Sherbrooke

**Keywords:** pediatric, ewing sarcoma, extraskeletal ewing sarcoma

## Abstract

Ewing sarcoma of the abdomen is a rare entity in pediatric oncology and represents a technical challenge both for surgeons and radiation oncologists. We document the case of a young female patient with primary disseminated, intraperitoneal Ewing sarcoma who after an excellent response to chemotherapy received preoperative whole abdominal-pelvic radiotherapy with good tolerance.

## Introduction

Ewing sarcoma is most commonly a bone tumor and involvement of the peritoneum has been rarely described. Peritoneal dissemination may occur via metastatic spread from the primary tumor, tumor spillage during surgical resection, or primary peritoneal disease with local spread [1]. In osseous Ewing sarcoma, the standard of care is a combination of chemotherapy and local treatment, usually surgery with or without postoperative radiotherapy (RT) or radiotherapy alone for patients with unresectable tumors. Given the very low incidence of primary intraperitoneal Ewing sarcoma, the treatment algorithm is not well established. For disseminated abdominal primary Ewing sarcoma, whole abdominopelvic radiotherapy (WAP-RT) has been described as part of a multimodality approach [2]. However, the therapeutic benefit and toxicity of RT in this setting is uncertain. The current Children’s Oncology Group (COG) AEWS0031 protocol recommends total abdominal-pelvic radiotherapy to a dose of 24 Gy in 16 daily fractions. We report the acute toxicity of WAP-RT in a patient with disseminated Ewing sarcoma of the peritoneum using volumetric modulated arc radiotherapy (VMAT).

## Case presentation

**Figure 1:**
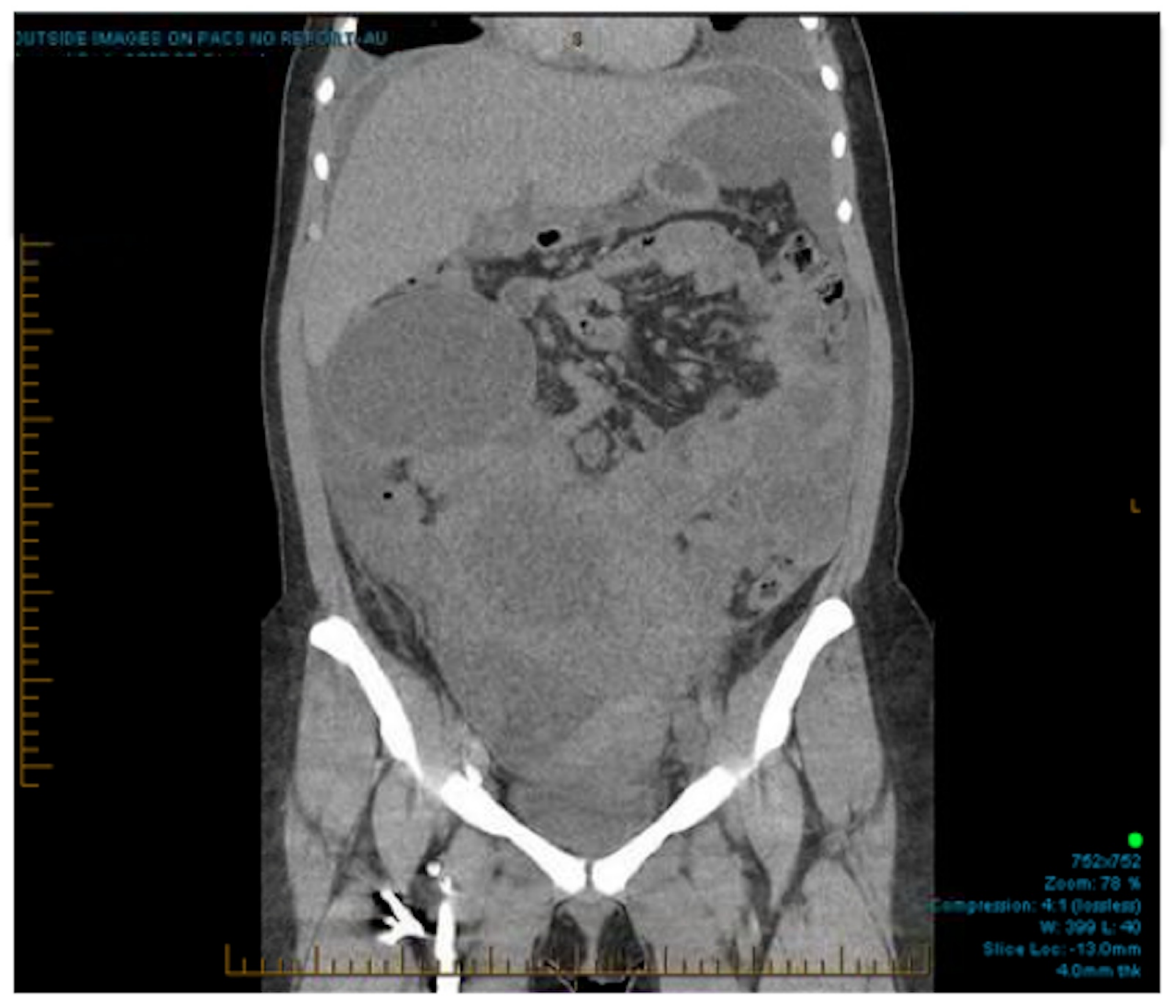
Staging CT scan. Coronal CT scan showing multiple abdominal agglomerated masses with cystic components revealing evidence of peritoneal carcinomatosis.

**Figure 2:**
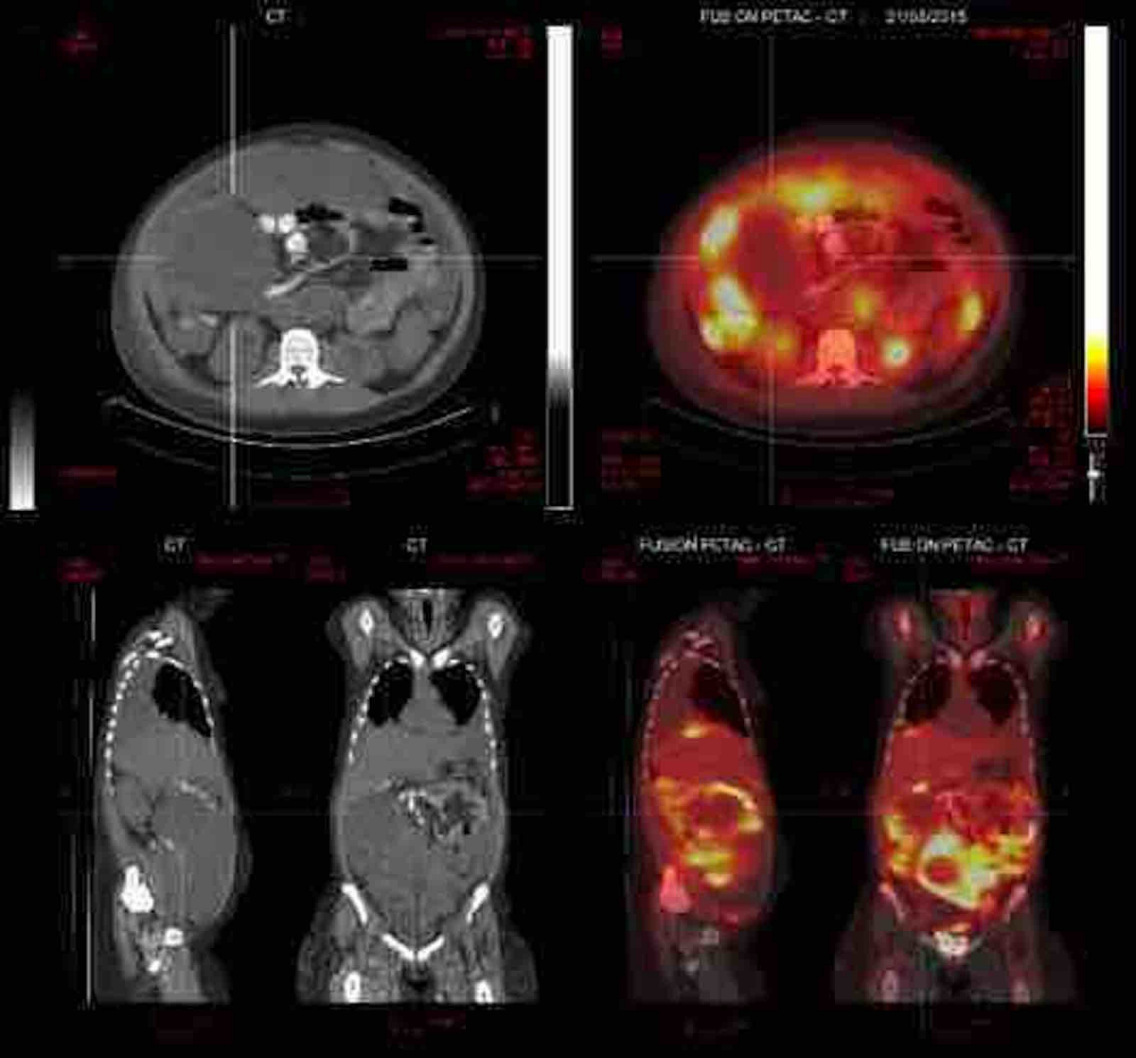
Staging PET-CT scan. PET-scan before the first cycle of chemotherapy showing multiple areas of increased metabolic activity in the abdomen and pelvis (maximum SUV 11.6) along with bilateral pleural effusions.

A 16-year-old female patient presented in May 2015 with dyspnea and a mass in the periumbilical area. An ultrasound-guided biopsy of a peritoneal implant confirmed a diagnosis of Ewing sarcoma, with a FLI-EWSR1 translocation. Computed tomography (CT) and Positron Emission Tomography (PET) staging identified multiple areas of increased metabolic activity in the abdomen and pelvis (maximum SUV 11.6) along with bilateral pleural effusions (Figures 1-2). Pleurocentesis was negative for malignant cells.

She was started on chemotherapy as per the AEWS0031 protocol with vincristine/doxorubicin/cyclophosphamide (VAC) alternating with Ifosfamide / VP16 chemotherapy (IE) on a two-week interval schedule, and completed her 8th cycle of treatment with good tolerance apart from expected hematologic toxicity, weight loss (<10% body weight), mild neuropathy, GERD, and constipation. She did not require unscheduled hospitalizations.

**Figure 3:**
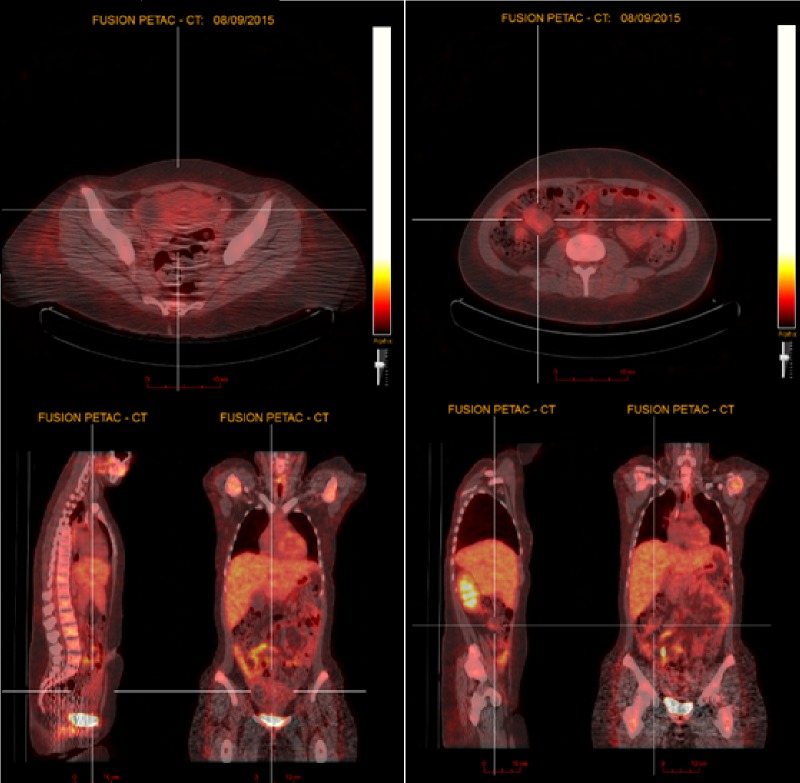
Re-staging PET-CT scan PET-scan after six cycles of chemotherapy showing excellent response with only two residual masses: one in the right flank with an SUV of 2.3, and a second in the pelvis with an SUV of < 2.

**Figure 4:**
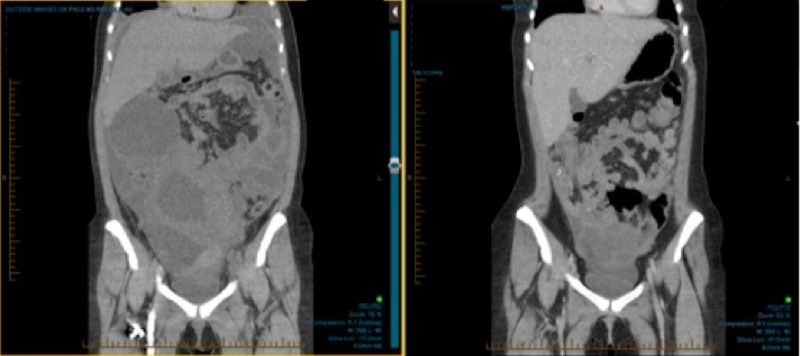
Coronal CT scan prior and after chemotherapy. Comparison between staging CT scan and after the 6th cycle of chemotherapy, revealing significant regression with two residual lesions: one in the right flank measuring 4.5 cm in greatest dimension and one in the pelvis measuring 10.7 cm in greatest dimension.

Repeat CT and PET scans after six cycles of chemotherapy in September 2015 showed an excellent radiologic and metabolic response. There were only two residual masses: one in the right flank that measured 4.5 cm in greatest dimension with an SUV of 2.3, and a second in the pelvis that measured 10.7 cm in greatest dimension with an SUV of < 2 (Figures 3-4). Our center’s multidisciplinary tumor board was consulted and the available literature was reviewed. The consensus was to offer the patient preoperative RT to the whole abdomen and pelvis to achieve downsizing and improve the likelihood of complete resection.

**Figure 5:**
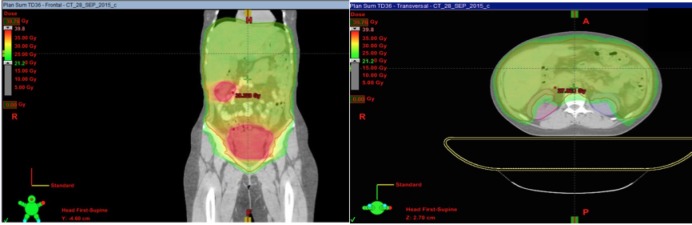
Total dose distribution. Left: Color wash coverage to the peritoneal cavity with homogeneous distribution on a dose of 24 Gy (light green) and a boost of 36.3 Gy delivered to the residual lesions (red).  Right: acceptable dose distribution in both kidneys.

The patient underwent WAP-RT with concurrent and subsequent boosts to the two residual, metabolically active lesions. Given that the patient measured 1.82 m (weight 110 kg, BSA 2.31 m^2^) and that her abdominal craniocaudal extent was 53 cm, her treatment plan was a technical challenge. It became apparent that field junctioning with a linear-accelerator based treatment would be required. Another challenge was to protect her kidneys while maintaining coverage to the entire intraperitoneal cavity since renal tolerance is a known limiting constraint [3]. A total abdominopelvic RT dose of 24 Gy with a boost of 12.3 Gy to the two residual masses was prescribed, for a total dose of 36.3 Gy to the masses (Figure 5).

**Figure 6:**
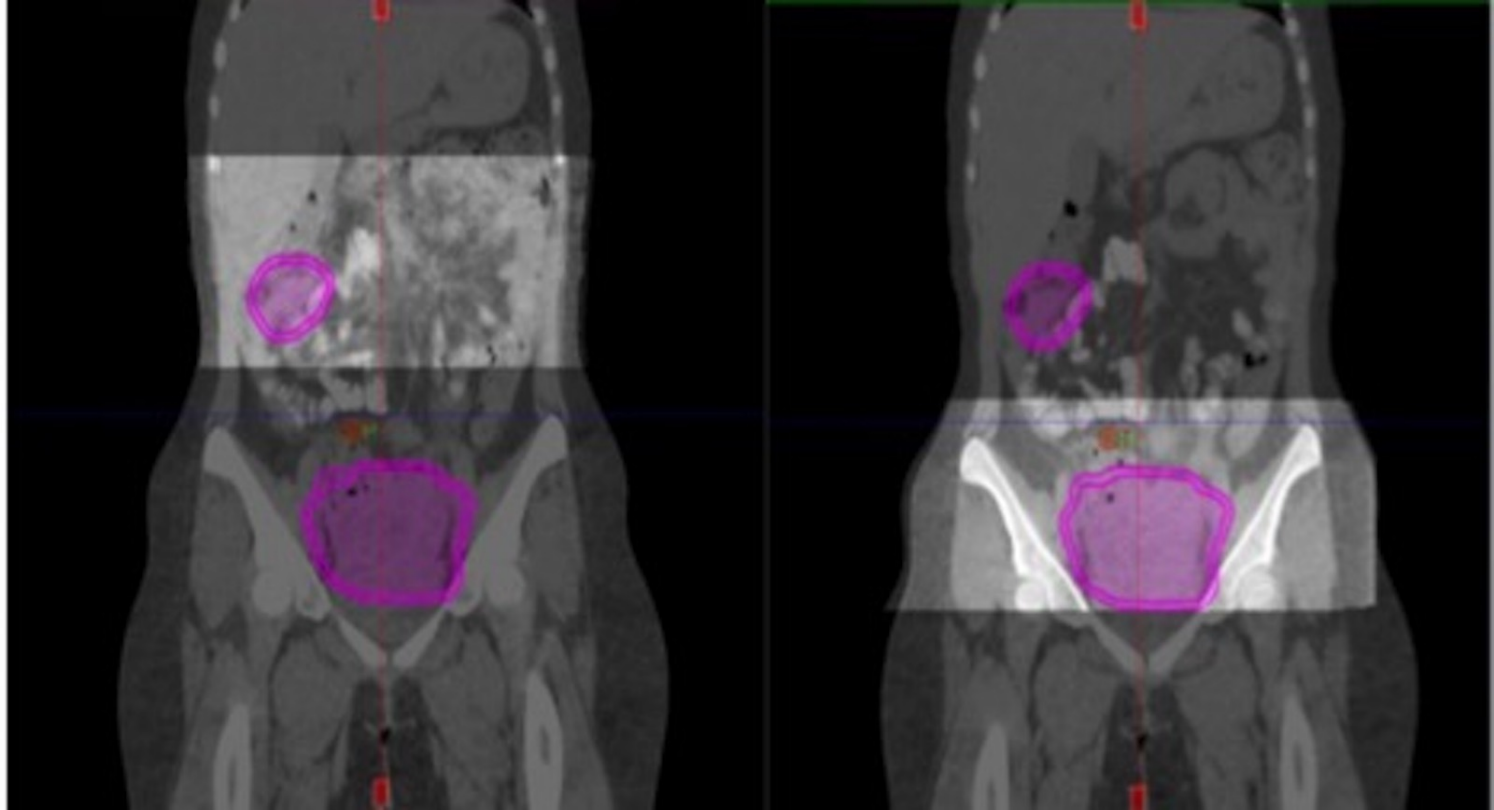
Superior and Inferior abdominal Cone-Beam CT during radiation treatment.

The patient was prescribed Ondansetron to be taken before and after RT for antiemetic prophylaxis. Two (inferior and superior abdominal) daily kilovoltage cone-beam computer tomography scans (CBCT) were performed for image guidance on a TrueBeam linear accelerator (Varian Medical Systems) (Figure 6). Target coverage was verified daily with CBCT.

Toxicity was analyzed at least once weekly during RT. Treatment was well tolerated and delivered without interruptions. As per the National Cancer Institute Common Terminology Criteria for Adverse Events v4.3 (CTCAE) [4], the patient experienced Grade 1 fatigue, Grade 1 dermatitis, Grade 2 dyspepsia, Grade 2 diarrhea, and Grade 2 genitourinary toxicity. They were managed conservatively; her dyspepsia was relieved with twice-daily proton pump inhibitor (Pantoprazole), and her diarrhea was controlled with dietary restriction and piperidine derivate (Loperamide). In addition, the patient had Grade 3 hematologic toxicity attributed to her systemic therapy requiring two platelet and two red blood cell transfusions. There was no alteration of her renal function. After the beginning of RT, the 9th cycle of chemotherapy was postponed by three days because of thrombocytopenia, and the 10th cycle was also postponed by two weeks for the same reason. The patient underwent complete surgical resection without complications seven weeks (delayed due to complicated febrile neutropenia and prolonged thrombocytopenia) following completion of RT.

All the residual masses were removed. The peritoneal lavage cytology was negative for malignant cells and revealed only chronic inflammatory and reactive cells.

## Discussion

There are few studies reporting the treatment of peritoneal Ewing sarcoma [2,5-7]. In a SEER review conducted by Applebaum et al. [5], patients with Extraskeletal Ewing’s Sarcoma (ESE) were found to have worse prognoses in the short term (in the first two years). The authors proposed that these patients might need treatment strategies that include multimodality treatment with surgery, radiotherapy, and chemotherapy. Patients who had a complete resection appear to face better outcomes.

For example, Ahmad et al., based on their review of twenty-four patients with ESE, concluded that wide resection was a major determinant factor that impacted overall survival [6]. The quality of radiotherapy was also important.

Kinsella et al. [7] analyzed a series of eleven patients with ESE; only one who had retroperitoneal disease received radiation treatment but failed locally. However, the author disclosed a potential inadequacy of the radiotherapy portals.  

In our patient, we used preoperative chemoradiotherapy with the goal of maximizing the chance of achieving a complete resection. An IG-IMRT technique confirmed on a daily basis the accuracy of treatment.

## Conclusions

We present a possible approach for disseminated, intra-abdominal Ewing sarcoma that includes whole abdominal-pelvic radiotherapy. Our patient was treated to a dose of 24 Gy plus a boost to residual masses; the acute toxicity was acceptable and was followed by complete resection without complication.
